# A Moving Residual Limb: Botulinum Toxin to the Rescue

**DOI:** 10.1515/tnsci-2020-0006

**Published:** 2020-02-29

**Authors:** Marie-Michèle Briand, Mathieu Boudier-Réveret, Xavier Rodrigue, Geneviève Sirois, Min Cheol Chang

**Affiliations:** 1 Department of Physical Medicine and Rehabilitation, College of Medicine, Yeungnam University 317-1, Daemyungdong, Namku, Taegu 705-717, Republic of Korea; 2 Institut de réadaptation en déficience physique de Québec, Québec, Canada; 3 Department of Physical Medicine and Rehabilitation, Centre hospitalier de l'Université de Montréal, Montreal, Canada

**Keywords:** Amputation stump, myoclonus, movement disorders, Botulinum toxin

## Abstract

Movement disorders post-amputation are a rare complication and can manifest as the jumping stump phenomenon, a form of peripheral myoclonus. The pathophysiology remains unknown and there is currently no standardized treatment. We describe the case of a 57-year-old male with unremitting stump myoclonus, starting one month after transtibial amputation, in his residual limb without associated phantom or neurological pain. The consequence of the myoclonus was a reduction in prosthetic wearing time. Failure to respond to oral medication led us to attempt the use of botulinum neurotoxin Type A injections in the involved muscles of the residual limb. Injection trials, over a two-year period, resulted in an improvement of movement disorder, an increased prosthetic wearing time and a higher satisfaction level of the patient. Injection of botulinum toxin type A should be considered as an alternative treatment for stump myoclonus to improve prosthetic wearing time and comfort.

## 1 Introduction

Jumping stump is a rare post-amputation complication. It has been described as a form of peripheral myoclonus which is characterized by sudden, brief and sometimes repetitive muscle contractions [1]. It has been reported after peripheral nerve trauma like in amputation [1].

No guideline or standardized treatment exist for jumping stump or peripheral myoclonus. It seems that oral medication, usually benzodiazepines, has been previously successful but mostly with central aetiologies, and has been disappointing with peripheral myoclonus [2]. Localized injections with botulinum neurotoxin type A (BoNT-A) [3] and type B [4] have been reported as the most promising treatment.

## 2 Case report

Here, we describe the case of a 57-year-old man diagnosed with hypertension and admitted in June 2014 for a below-knee amputation due to an infected post-traumatic aneurysm of the right popliteal artery in which distal limb salvage was impossible. One month after amputation, the patient noted repetitive muscle contractions in his stump. The contractions were continuous in nature and caused discomfort during the use of his prosthesis but were not painful. They did not wake him up at night. Pharmacological therapy was attempted and included sequentially carbamazepine, levetiracetam, pregabalin and clonazepam without satisfactory results.

In May 2015, when initially seen by the treating physiatrist, eleven months post- amputation, the patient reported constant involuntary movements of his residual limb. He also reported that phantom pain had been present since amputation but improved with the use of his prosthesis. However, his wearing time was limited by discomfort due to involuntary movement and pressure sensation when wearing his prothesis. Myoclonus and phantom pain did not appear to be associated with one another, neither with temperature, voluntary movement, alcohol consumption, nor noise. The patient’s medication list included: acetylsalicylic acid, clonazepam, diclofenac, diltiazem, hydromorphone contin, perindopril, pantoprazole, as well as fluticasone and salmeterol combination puffer.

On physical examination, rhythmic and involuntary movements were visible in the residual limb (Video 1 – Supplementary material). Tinel’s sign was present at the fibular and tibial nerves, but no trigger points for myoclonus were identified. Muscles of the lateral and posterior compartments appeared to be affected by the myoclonus. Neurological examination was otherwise non-contributory including cranial nerves and cerebellar examination. Blood tests were unremarkable. Normal nerve conduction studies were measured on the contralateral leg to rule out neuromuscular disease. Electromyogram (EMG) was conducted on the residual limb. No spontaneous waveforms were found in the rectus femoris, adductor longus, psoas major and biceps femoris following needle examination. However, spontaneous waveforms of normal amplitude and duration were seen in the tibialis posterior, peroneus longus and brevis as well as the gastrocnemius; which were the muscles suspected on being affected by myoclonus based on the physical examination.

In July 2015, sonography of the residual limb showed tibial (5 x 8 x 10 mm) and common fibular (5 x 7 x 7 mm) neuromas. Myoclonus was identified predominantly in the posterior muscle group and to a lesser extent in the lateral muscle group. Neither the semitendinosus nor the semimembranosus showed myoclonus. Sonography-guided bupivacaine (0.5%, 1 mL) and dexamethasone (10 mg) block of the sciatic nerve 10 cm proximal to the femorotibial line was performed. An immediate 75% reduction in myoclonus frequency was reported post-lidocaine injection. This improvement lasted for approximately three weeks with subsequent recurrence. Two months later, in September 2015, a second sonography-guided lidocaine (1%, 2 mL) and dexamethasone (10 mg) block, distal to hamstring innervation, was attempted and resulted in complete cessation of the myoclonus for two days.

Botulinum neurotoxin type A injection was attempted for the first time in February 2016 and was continued over a two-year period. Six appointments were necessary to find the right target muscles (e.g. medial and lateral gastrocnemius, tibialis anterior and peroneus longus and brevis) and dosage (see Table 1 for detailed injection protocol and results). EMG guidance was the method used to select muscles and sonography guidance was sometimes used to confirm muscle identification given the altered anatomy post amputation. To evaluate the success of this therapeutic approach, we focused on the improvement of the movement disorder, the prosthesis wearing time (in hours/day) and the level of satisfaction of the patient (with a scale of 0 to 10 – 0, total dissatisfaction and 10, total satisfaction). Video 1 (Supplementary material) also presents the patient’s residual limb at the end of the two-year follow-up.

**Table 1:** Botulinum type A (BoNT-A) injections table. *: injections were done with sonography guidance, if no * EMG guidance was used. Satisfaction level scale: 0 means total dissatisfaction and 10 means total satisfaction. Botox^®^ (Allergan, USA) was the injected product.

Injections	Time	Total units (U)	Efficacy period (weeks)	Prosthetic wearing time (hours/day)	Satisfaction level (0-10 scale)
BoNT-A injection 1	February 2016	**100**	2	8	3
		Medial gastrocnemius - 25			
		Lateral gastrocnemius - 50			
		Peroneus longus - 15			
		Peroneus brevis - 10			
BoNT-A injection 2	May 2016	**200**	2	8	3
		Medial gastrocnemius - 50			
		Lateral gastrocnemius - 75			
		Peroneus longus - 25			
		Peroneus brevis - 25			
		Tibialis anterior - 25			
BoNT-A injection 3*	November 2016	**300**	8	8	3
		Medial gastrocnemius - 75			
		Lateral gastrocnemius - 75			
		Peroneus longus - 50			
		Tibialis anterior - 100			
Total year injection: 600 U					
BoNT-A injection 4	February 2017	**300**	8	16	4
		Medial gastrocnemius - 75			
		Lateral gastrocnemius - 75			
		Peroneus longus - 50			
		Tibialis anterior - 100			
BoNT-A injection 5	April 2017	**300**	6	16	4
		Medial gastrocnemius - 75			
		Lateral gastrocnemius - 100			
		Peroneus longus - 25			
		Peroneus brevis - 25			
		Tibialis anterior - 75			
BoNT-A injection 6	July 2017	**400**	10	16	7
		Medial gastrocnemius - 100			
		Lateral gastrocnemius - 125			
		Peroneus longus - 100			
		Peroneus brevis - 75			
Total year injection: 1000 U					
Evaluation post-injection	September 2017		8	16	9
					

**Ethical approval:** The research related to human use has been complied with all the relevant national regulations, institutional policies and in accordance the tenets of the Helsinki Declaration, and has been approved by the authors’ institutional review board or equivalent committee.

**Informed consent:** Informed consent has been obtained from patient included in this study

## 3 Discussion

In this case report, the painless jumping stump responded successfully to BoNT-A injections after having failed other more conventional pharmacologic treatments.

Botulinum toxin, which prevents the release of the neurotransmitter acetylcholine at the axon ending of the neuromuscular junction, acts to denervate muscles and has been successfully used in other types of myoclonus [5, 6]. However, the efficacy is often temporary, because recovery of muscle innervation occurs through proximal axonal sprouting and muscle reinnervation by formation of new neuromuscular junctions. Increased total dosage of botulinum toxin was necessary firstly to establish a day-lasting prosthetic wearing time, and secondly to relieve the pressure sensation present in the donning period. The patient clearly differentiated phantom pain (which was improved wearing his prosthesis) from the pressure sensation, but atypical neurologic pain was not excluded for this sensation and neuromas were our main diagnostic hypothesis to explain the abnormal movement.

This association between jumping stump and neuroma was suggested by Tyvaert et *al*. [7] and Buntragulpoontawe et *al*. [8] in case reports, and they both described how touching or tapping the neuromas provoked painful involuntary movement which was not present in our case. They chose to manage the painful involuntary movement with lidocaine injection [7] and with phenol injection [8] which have been both reported as successful. In our case, lidocaine injection was tried twice, and each time lasted longer than it should have to consider the test as conclusive. Phenol injection was not easily accessible in our facilities and was discarded for this technical reason. Our clinical approach did not confirm or infirm the association between neuroma and jumping stump. This was also a reason why surgery was not performed, in addition to the high likelihood of surgery-related morbidities and neuroma recurrence even though it is a well-established treatment for neuromas [9].

To date, there are no consensus-based best practice recommendations to treat peripheral myoclonus or jumping stump. Carbamazepine has been suggested as a therapeutic option [10], but this approach failed in our case. Previous success with BoNT-A injections has been described in residual limbs [3, 7] with movement disorders. Dave et *al*. (2010) [3] injected 75 U of BoNT-A into the tibialis anterior and 25 U into the gastrocnemius with EMG guidance. At one-month follow-up visit, the residual limb abnormal movement, identified as myokymia by EMG, was reduced by 70% but the patient was subsequently lost to follow-up. Tyvaert et *al*. [7] reported, in a single case, the use of 30 U BoNT-A injection around the scar of the shoulder disarticulation amputation. It led to the disappearance of peripheral myoclonus one week after the injection and to a 14-week period of resolved involuntary movement but only reduced pain. No further follow-up was reported. However, success using saline injections has been reported to be as high as BoNT-A injections to treat chronic functional movement disorders which include a wide variety of diagnosis [11].

In our case, follow-up was mandatory because of recurring symptoms. Due to the lack of literature on the subject, conservative approach was adopted and dosage as well as target muscles were eventually adjusted. We persisted with this treatment because periods of relief were clearly associated with BoNT-A efficacy period even if a placebo response cannot be completely ruled out. In our point of view, sonography guidance could be a useful tool to identify muscles in the stump due to the lack of traditional anatomical landmarks given the unique anatomy of each residual member. In conclusion, it is clear that a better understanding of the pathophysiology of peripheral myoclonus, the so-called jumping stump, could help to choose the best therapeutic approach. It is possible that BoNT-A addressed the consequences rather than the cause of this movement disorder. Nonetheless, it was sufficient to achieve the main therapeutic goals; reduction of myoclonus, increase in prosthesis wearing time and high satisfaction level without side effects.
